# Internet Digital Economy Development Forecast Based on Artificial Intelligence and SVM-KNN Network Detection

**DOI:** 10.1155/2022/5792694

**Published:** 2022-06-20

**Authors:** Jianru Fu, Xu Zhou, Guoping Mei

**Affiliations:** ^1^School of Finance, Jiangxi Normal University, Nanchang, Jiangxi 360100, China; ^2^Management Science and Engineering Research Center, Jiangxi Normal University, Nanchang, Jiangxi 360100, China

## Abstract

The development and spread of Internet technology have made it easier to find web servers. People can browse various websites to shop or pay for living expenses, which brings great convenience to life, but as a result, Internet security problems continue to appear. This article is based on a detailed theoretical analysis of mainstream algorithms, making an analysis of web logs which is of great significance and practical value. In addition, through reasoning analysis, technical support is provided for improving the weight factor of the KNN (*K*-nearest neighbor) algorithm, and the literature research method of the SVM-KNN hybrid algorithm and the KNN classifier is proposed. This paper conducts a detailed theoretical analysis based on the mainstream algorithms that are widely used in the current classification technology and integrates the mainstream classification algorithms in real-life applications and popularization, selecting the support vector machine and KNN calculation method. In the digital economy development model, although China has a large number of netizens, obvious late-comer advantages and institutional advantages as a guarantee, due to the constraints of two key factors, capital and technology, a series of social problems have also arisen. During the transformation of the digital economy, prominent digital security issues, high-risk vulnerabilities, and increasing number of cyber-attacks, along with uneven data quality levels and lagging laws and regulations, have brought many challenges and obstacles.

## 1. Introduction

As more and more business and social activities shift from offline to online, the security of web services becomes more and more important as a branch of network security [1]. Abnormal behavior analysis, namely intrusion detection, is a new network security mechanism used to detect, prevent, and exclude unauthorized users from unauthorized access to communications or computer networks [2, 3]. Therefore, abnormal behavior analysis is an important research topic in network security. The server has the characteristics of remote access and a large number of security vulnerabilities [4]. Hackers can use these vulnerabilities to damage the web server, collect secrets from the database, and even interrupt or replace the web server. Because web logs record the behavior of users accessing the network, analyzing web logs is one of the most effective ways to identify abnormal user behavior [5]. Through derivation and analysis of the fitting principle of the traditional SVM-KNN hybrid algorithm and related literature research on the improvement of the KNN classifier, a weight factor improvement plan for the KNN algorithm is proposed [6]. Based on the above fitting theory and improvement plan, a support vector machine classification is proposed. The SVM reconciliation weighed KNN algorithm based on the mutual fitting of the KNN classifier and the SVM classifier. With the continuous development of science and technology, data and information in various fields and research disciplines are growing at an exponential rate [7, 8]. Especially in recent years, in order to find potential users in various industries in the market, more and more daily information and data will be reserved for users, the rational use of data and information has become more and more important in daily production activities, and the application requirements for data and information are spreading in all aspects of people's lives [9]. With the advent of the era of big data, the reasonable division and application of data information can make people's production and life more convenient and efficient [10]. How to efficiently and meaningfully use massive amounts of data is a problem worthy of research and discussion at the moment. So the data classification algorithm came into being. Since the outbreak of the international financial crisis in 2008, the global economy has officially entered a stage of adjustment. The old and new economic development models have alternately changed. The specific manifestation is the continuous sluggish development of traditional economic models and the emergence of digital economic models based on Internet technology. The digital economy is in a period of rapid development. Smart machines have become a new production tool, data have become a new production factor, and information networks have become a new infrastructure. It is driving the digital economy towards a networked connection, data description, and intelligent production. It is a new era of integrated development of the industrial Internet.

## 2. Related Work

Some research works proposed a KNN clustering model based on density peaks, which can detect attacks more effectively and introduce density into KNN [11]. This process does not require many parameters, and the iterative process is based on density. Literature proposed a new Vervis intrusion detection model, in which a new data mining classification method is combined, that is, as an alternative to the Vervis network using the K2 algorithm [12]. Some research proposed an extreme learning time as a basic classification method and a random character space assembly method with bat calculation method as a pruning method [13]. Literature proposed a boost enhancement algorithm, which is based on the adaptive combination of multiple delimiters to detect accuracy, using a smoothing method to ensure the stability and detection efficiency of the classification system [14]. Some research introduced two new hybrid intrusion detection technologies, namely gravity-based search and combined search based on GS and particle swarm optimization. These two technologies have been successfully applied to artificial neural network models and effectively detect anomalies. Some research proposed two gradient descent algorithms for constructing a neural-network-based intrusion detection system, namely a momentum-based back propagation algorithm and a momentum-based adaptive gain back propagation algorithm. Some research applies decision tree algorithm and KNN algorithm to the classification of soil data and obtains the distribution of local soil components through data classification, which provides data reference for selecting the most suitable crops for planting and provides local agricultural production. Other works use classification technology in the process of urban data discovery and apply the classification technology in data mining to the probability of occurrence of urban illegal incidents, which played a role in preventing crimes to a certain extent and improved the ability to supervise areas with high crime rates. Some scholars use data mining technology in the cost forecast of engineering projects which, to a certain extent, improves the efficiency of engineers' overall planning of the project, as well as the project's construction cost expectations. IBM uses data mining technology to provide communication companies in many North American and European countries with a relatively complete business system development plan and provides huge support for communication services in the neighborhood of market analysis, customer decision-making, and commercial marketing.

## 3. Artificial Intelligence and SVM-KNN Network Detection

### 3.1. SVM-KNN Algorithm

**Figure 1:**
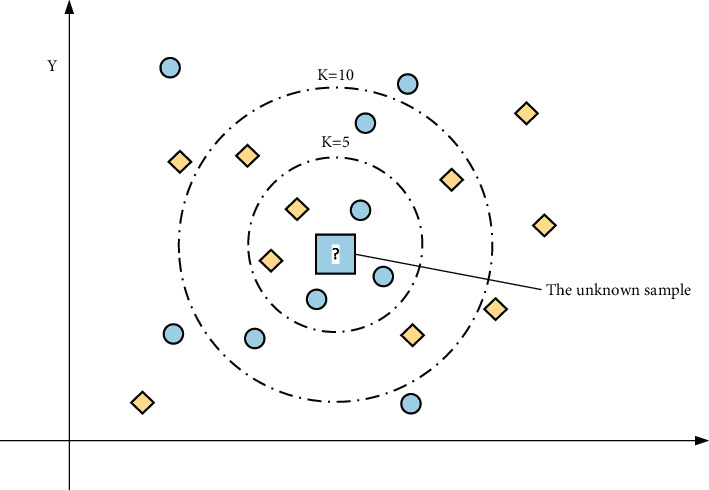
KNN method interpretation.

KNN classification algorithm is one of the classic algorithms in the field of machine learning, and it is one of the statistical learning methods based on the test samples proposed by Calibration and Hodges in the 1950s. Generally, the *K* value is taken in 10 steps, and the *K* value is selected with relatively high accuracy. In addition, when using the KNN minimum neighborhood algorithm to classify unknown samples, there is no need to compare samples, and by processing the values, the distance between the new sample to be classified and the training sample can be calculated. Due to the actual situation of the data sample, each data sample may have one or more characteristics. Therefore, the “finite plane” assumed when classifying the data may be three-dimensional or even multifaceted. The basic principle of calculating the *K* value distance is the same as that of the plan, as shown in Figure 1.

In the mathematics category, there are the spatial distance calculation formula used by the KNN algorithm, and most KNN algorithms use the distance formula. Taking plane coordinates as an example, the Euclidean distance formula for calculating the distance between two points in plane coordinates is as follows:

(1) $$\begin{array}{c} \rho =\sqrt{{\left(x_{2}-x_{1}\right)}^{2}+{\left(y_{2}-y_{1}\right)}^{2}}. \end{array}$$

The actual calculation is the distance between the point (*x*_1_, *y*_1_) and the point (*x*_2_, *y*_2_), which is expanded into a multifaceted space. The formula is

(2) $$\begin{array}{c} d\left(x,y\right)=\sqrt{{\left(x_{1}-y_{1}\right)}^{2}+{\left(x_{2}-y_{2}\right)}^{2}+\cdots +{\left(x_{n}-y_{n}\right)}^{2}}\\ =\sqrt{\sum_{i=1}^{n}{\left(x_{i}-y_{i}\right)}^{2}}. \end{array}$$

**Figure 2:**
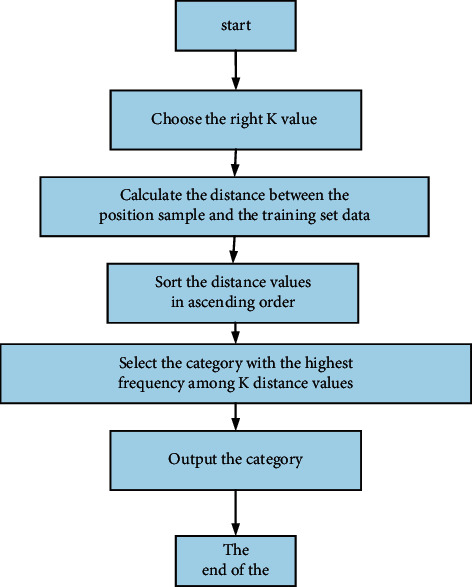
KNN algorithm flowchart.

Based on the above formula, obtain the distance between the sample point to be classified and each training sample point, and arrange the distance value to select the closest *K* distance:

1. Obtain the distance value from the sampling point to be measured to the training concentration sampling point through the distance formula.
2. The calculated intaglio interval should be arranged in ascending order.
3. Choose a small *K* point.
4. Determine the frequency of each type in the *K* point.
5. Return the classification with the highest frequency. The KNN algorithm process is as shown in Figure 2.

This section constructs the mathematical model of the KNN algorithm and uses the mathematical formula to express how the KNN classifier performs classification. The principle of the formula is as follows:

(3) $$\begin{array}{c} y_{t}=\underset{c\in \left\{c_{1},c_{2},\ldots ,c_{m}\right\}}{\arg\max}\sum_{x_{t}\in N\left(x_{T},k\right)}E\left(y_{i},c\right). \end{array}$$

Among them, the unknown samples are *xi* and *yi*, and *m* is the number of types of the total sample data.

(4) $$\begin{array}{c} E\left(a,b\right)=\left\{\begin{array}{cc} 1, & \text{if }\left(a=b\right),\\ 0, & \text{else}. \end{array}\right.. \end{array}$$

N(x, k) is the closest set of *x* in the sample data. The reasons are as follows:

(5) $$\begin{array}{c} p{\left(c_{j}\right)}_{\left(x_{t},k\right)}=\sum_{x_{t}\in N\left(x_{t},k\right)}\frac{E\left(y_{i},c_{j}\right)}{k}. \end{array}$$

Among them, *p*(*c*_1_) (*xt*, *k*) is the probability that the sample belongs to class c1. According to this formula, it can be simplified as follows:

(6) $$\begin{array}{c} y_{t}=\arg\max\left\{p{\left(c_{1}\right)}_{\left(x_{t},k\right)},p{\left(c_{2}\right)}_{\left(x_{t},k\right)},\ldots ,p{\left(c_{m}\right)}_{\left(x_{t},k\right)}\right\}. \end{array}$$

It can be seen from the above equation that the mathematical model is a sample type to be measured with a priori probability. Vector algorithm is a model recognition method based on computational learning theory. Statistical theory is based on statistics of small data sets. It is developed based on the research background. Support vector machines have obvious performance advantages. It can better solve the recognition problems of nonlinearity, small data sets, and high latitudes in pattern recognition. Now it is widely used in face recognition, intrusion detection, text classification, and credit rating evaluation field.

**Figure 3:**
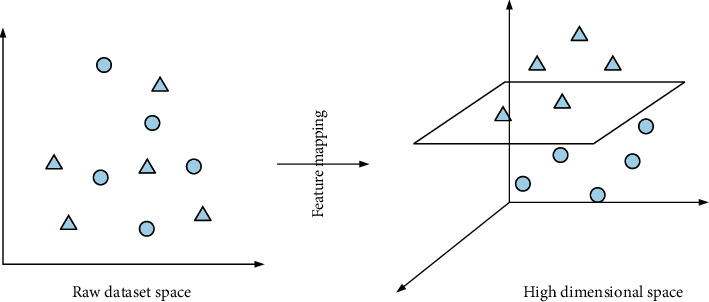
Nonlinear mapping.

The latent matter calculation method is a model identification method. It creates a theoretical framework based on statistical learning theory. The model was outlined in the late 1990s and has been focusing on potential research. The classification process of support vector machines is to find the interface to classify different categories. The samples are divided, the multicategory problem is converted into a one-to-one or one-to-many convex binary classification problem, and the quadratic programming problem is solved. It has unique advantages when solving support vector nonlinear problems and can be transformed into nonlinear problems in low-dimensional data set space through the principle of its mapping. The high-dimensional feature is to decompose the space into nonlinear problems, as shown in Figure 3.

In order to obtain the best superslicing solution, it is necessary to internally load the sample data concentration data into the feature space. The internal load between the sample data not only is the same as the internal load between the original samples but also reduces the computational complexity to a certain extent. The optimal hyperplane is also based on support vectors. The efficiency of the classification algorithm depends on the number of vectors; it has no direct relationship with the dimension of the sample data set and has a good ability to delete and analyze high-dimensional feature data sets. SVM converts nonlinear or low-dimensional data problems into high-dimensional space and converts the nonlinear problem into an approximately linear optimal hyperplane. Therefore, the global optimal solution is obtained by solving the optimal hyperplane, which solves the problem in back propagation (BP), the local extremum problem in neural networks.

**Figure 4:**
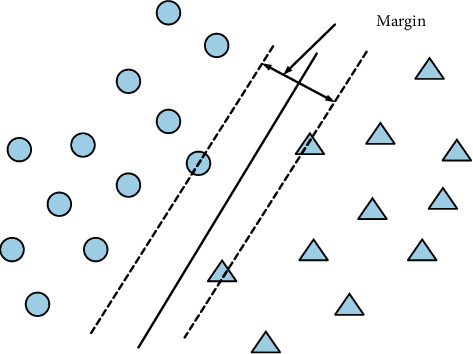
SVM optimal hyperplane schematic diagram.

The SVM algorithm is proposed based on the research summary of the linearly separable optimal classification hyperplane. The circle and the triangle represent two different training sample values, and *L* is the line separating the two different sample values. Separate *L*1 and *L*2, respectively, and the separation between *L*1 and *L*2 is called two kinds of separation, as shown in Figure 4.

The statistical learning theory should reliably separate the optimal dividing lines L1 and L2 to reduce the experience risk and the separated data, so as to obtain a solution. The inner product formula of the best hyperplane is as follows:

(7) $$\begin{array}{c} \omega \cdot x+b=0. \end{array}$$

If the discriminant functions are integrated and combined so that the dividing line can classify data according to accurate categories, the following expressions must be satisfied:

(8) $$\begin{array}{c} y_{i}\left[\omega \cdot x+b\right]-1\geq 0.i=1,2,\ldots ,n. \end{array}$$

That is equivalent to

(9) $$\begin{array}{c} \left\{\begin{array}{cc} \left(w^{T}\cdot x_{i}\right)+b\geq 1, & y_{i}=+1,\\ \left(w^{T}\cdot x_{i}\right)+b\leq 1, & y_{i}=-1. \end{array}\right.. \end{array}$$

The interval between the two types of data is 2, 1, and 4. Then, the value should be the minimum value in order to increase the gap between the two data types.

(10) $$\begin{array}{c} \left\{\begin{array}{c} \min\varphi \left(w\right)=\frac{1}{2}{\left\|w\right\|}^{2},\\ \text{s.t }y_{i}\left[\left(w^{T}\cdot x_{i}\right)+b\right]-1\geq 0,i=1,2,\ldots ,N. \end{array}\right.. \end{array}$$

The method to optimize the function by adding the quantity again is as follows:

(11) $$\begin{array}{c} \left\{\begin{array}{c} \min L\left(\alpha \right)=\frac{1}{2}\sum_{i,j=1}^{N}\alpha_{i}\alpha_{j}y_{i}y_{j}\left(x_{i}\cdot x_{j}\right)-\sum_{i=1}^{N}\alpha_{i},\\ \text{s.t }\alpha \geq 0,\;i=1,2,\ldots ,N,\\ \sum_{i=1}^{N}y_{i}\alpha_{i}=0. \end{array}\right.. \end{array}$$

Therefore, a simplified formula can be used to obtain the SVM classification.

(12) $$\begin{array}{c} f\left(x\right)=\mathrm{sgn}\left[\sum_{i=1}^{N}\alpha_{i}y_{i}\left(x\cdot x_{i}\right)+b\right]c. \end{array}$$

The above equation can solve linearly decomposable data. However, in most cases, the timetable is nonlinear and high dimensional. The kernel function *K* is added to the SVM classifier to solve the classification problem of the data set. The internal calculation result of the kernel function *K* can be in the equivalent high-dimensional timetable. The productivity calculation is equivalently replaced in the data space, and the formula is as follows:

(13) $$\begin{array}{c} K\left(x\cdot x_{i}\right)=\phi \left(x\right)\cdot \phi \left(x_{i}\right). \end{array}$$

The simultaneous formula can be obtained by Lagrange transformation:

(14) $$\begin{array}{c} f\left(x\right)=\mathrm{sgn}\left[\sum_{i=1}^{N}\alpha_{i}y_{i}\left(x\cdot x_{i}\right)+b\right]. \end{array}$$

How to choose the appropriate kernel function *K* in the trusted vector algorithm plays a key role in the classification accuracy and efficiency of the algorithm. Each kernel function has the advantages of dealing with and solving specific problems. Choosing the appropriate kernel function can greatly improve the classification accuracy of the classifier. Choosing the appropriate kernel function is the most important step in the SVM classifier, but the working principle of the kernel function *K* is as follows:

(1) There is a nonlinear data space sample set, and the nonlinear data in the initial data space in the nonlinear data space can be converted into a high-latitude space by using a mapping method.
(2) In the higher latitude space, the root linear calculation formula is selected and converted into root linear or linear dot product calculation.
(3) The asynchronous form of nonlinear sample data collection can be ignored, and if the kernel function satisfies Mercer's theorem, it will be damaged by internal cumulative calculations.
(4) Use the kernel function to solve the inner product problem and solve the initial space. In the vector budget method, several expressions are often used to select the kernel function *K*.
  (15) $$\begin{array}{c} K\left(x,x_{i}\right)={\left[\left(x*x_{i}\right)+1\right]}^{q}. \end{array}$$

Kernel function based on radial basis is as follows:

(16) $$\begin{array}{c} K\left(x,x_{i}\right)=\exp\left\{-\frac{{\left|x-x_{i}\right|}^{2}}{\sigma^{2}}\right\}. \end{array}$$

The difference from the RBF kernel function is that each basic function corresponds to a special vector, and the number of output vectors is the same as that determined by the calculation method itself.

(17) $$\begin{array}{c} K\left(x,x_{i}\right)=\tanh\left(v\left(x,x_{1}\right)+b\right). \end{array}$$

The vector algorithm is equipped with potential clutter key recognition equipment, determines the number of potential nodes, and also avoids local restrictions.

### 3.2. Network Anomaly Detection Based on Artificial Intelligence

**Figure 5:**
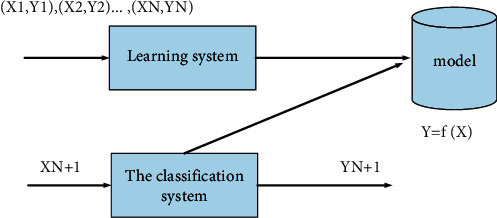
Schematic diagram of the classification problem.

The network intrusion detection behavior based on the above content can essentially be regarded as a classification problem. In other words, normal and abnormal behaviors can be classified from various network abnormal behavior data, specific attack methods can be determined from abnormal behaviors, and even unknown types of attacks can be detected. The process can be divided into three parts: learning system, model system, and classification system. First, the learning system uses a preprepared training data set to repeat the classification of output predictions. The task of classification is to determine the classification of the target object, as shown in Figure 5.

First introducing the linear regression method, the formula of the linear regression method is as follows:

(18) $$\begin{array}{c} z=\theta_{0}+\theta_{1}x_{1}+\theta_{2}x_{2}+\theta_{3}x_{3}\cdots +\theta_{n}x_{n}=\theta^{T}x. \end{array}$$

Among them, *x* is an independent quantity and 0 is a weighted number. This calculation method is used to learn a sample number instead of obtaining the best coefficient and then create a straight line to predict a new number. Broadly speaking, sigmoid function is derived and can be regarded as a linear regression model, and its theoretical regression formula is as follows:

(19) $$\begin{array}{c} h_{\theta }\left(x\right)=\frac{1}{1+e^{-z}}=\frac{1}{1+e^{-\theta^{T}x}}. \end{array}$$

Among them, in the *l* + *e* in (0 + 1) in the intermediate projection of the function result, it is used to represent the different probabilities of data belonging to a specific class, and it is very useful to use the demand probability for auxiliary analysis tasks, so you can use the S type function solving. The classification result input data *x* has each category of 1, and the probability of each category being 0 is as follows:

(20) $$\begin{array}{c} P\left(y=1|x;\theta \right)=h_{\theta }\left(x\right),\\ P\left(y=0|x;\theta \right)=1-h_{\theta }\left(x\right). \end{array}$$

Next, the optimal factor is obtained by calculating the solution of the loss function with the largest similarity score in probability theory, so as to maximize the classification effect.

(21) $$\begin{array}{c} P\left(y|x;\theta \right)={\left(h_{\theta }\left(x\right)\right)}^{y}*{\left(1-h_{\theta }\left(x\right)\right)}^{1-y}. \end{array}$$

Since the number of samples is independent of each other, their cascading probability distribution is as follows:

(22) $$\begin{array}{c} L\left(\theta \right)=\prod_{i=1}^{m}P\left(y^{\left(i\right)}|x^{\left(i\right)};\theta \right)=\prod_{i=1}^{m}{\left(h_{\theta }\left(x^{\left(i\right)}\right)\right)}^{y^{\left(i\right)}}*{\left(1-h_{\theta }\left(x^{\left(i\right)}\right)\right)}^{1-y^{\left(i\right)}}. \end{array}$$

Take the log likelihood function as

(23) $$\begin{array}{c} I\left(\theta \right)=\log\left(L\left(\theta \right)\right)=\sum_{i=1}^{m}\log\left({\left(h_{\theta }\left(x^{\left(i\right)}\right)\right)}^{y^{\left(i\right)}}\right)+\log\left({\left(1-h_{\theta }\left(x^{\left(i\right)}\right)\right)}^{1-y^{\left(i\right)}}\right). \end{array}$$

The method of maximum similarity is to solve the value of 0 when the maximum value is selected. It can be converted into a gradient descent algorithm and the factor can be solved by a simple conversion.

(24) $$\begin{array}{c} J\left(\theta \right)=-\frac{1}{m}I\left(\theta \right). \end{array}$$

Since this calculation method directly models the possibility of classification, there is no need to assume the data distribution in advance, thereby avoiding the problem caused by incorrect assumptions.

Assuming that there is a Ryu type in the training sample book, please enter the newly imported sample *X*, and the conditional probability of belonging to Ryu is PCx + *c*. According to Bees' theorem, the probability of verification is as follows:

(25) $$\begin{array}{c} P\left(c_{i}|x\right)=\frac{P\left(x,c_{i}\right)}{P\left(x\right)}=\frac{P\left(c_{i}\right)P\left(x|c_{i}\right)}{P\left(x\right)}. \end{array}$$

The current conditional probability is the combined probability of all attributes. For the convenience of calculation, the Bayesian classifier assumes that all attributes are independent of each other. In other words, it is assumed that attributes independently affect the classification results, but the probability of belonging to *c* can be written.

(26) $$\begin{array}{c} P\left(c_{i}|x\right)=\frac{P\left(c_{i}\right)P\left(x|c_{i}\right)}{P\left(x\right)}=\frac{P\left(c_{i}\right)}{P\left(x\right)}\prod_{j=1}^{d}P\left(x_{j}|c_{i}\right). \end{array}$$

It can be seen from the above that according to the simple Bayesian training process, the prior probability *p* can be guessed and the current conditional probability *p* can be predicted (*x* | *c*) for the classifier sample regression.

The algorithm close to *k* is an atypical parameter model algorithm. The sample is stored and trained in this step, and when a new sample is input, it will be intensively trained according to a certain distance measurement method. You can also find *k*, the most similar sample, and then input the type label of the new sample from the *k* most “neighboring” samples, or as the classification label of other samples, and train the sample to *k*. According to weighing the perspective distance, the closer the distance, the greater the weight of the sample.

So far, there are few studies on analyzing abnormal web behavior, but if you look at feature engineering, there is no systematic detailed explanation, and statistical features of related categories cannot be used in a single-layer classification model, which limits the detection efficiency. Traditional classification algorithms include logistic regression algorithm, support vector machine algorithm, naive Bayes algorithm, and KNN algorithm. For the logistic regression model, when the feature space is relatively large, the expression effect of the model is not very good, so it is easy to observe the consensus phenomenon; when there are many variables, the classification efficiency of the vector group is supported by the time and time of the *K* neighborhood model. The spatial density is relatively high and requires a long driving time, so this method cannot satisfy both low variance and low deviation at the same time, as shown in Table 1.

First, the artificial neurons of the sensory organs need to be explained. In the network structure in the figure, the input information is first considered, and then the sensors of the first layer determine and output relatively simple results; the sensors of the second layer determine the failure results of the first layer, and the sensor is better than the first layer. The perceptron is more complex and makes decisions more abstractly; the third layer is used to make more complex decisions, so the multilayer network of sensory organs participates in complex decisions. In this network structure, these three layers can be called input layer, nested layer, and output layer, respectively. In the neural network model, connection weights and deviations are very important. They constitute the structure of the entire neural network and the neural network model. It is used to determine the response method.

The neural network model has made a preliminary attempt in each area and achieved certain results. The main computational characteristics of the neural network are as follows:

1. Parallel and distributed processing capabilities: neural networks have a high degree of parallel structure and implementation capabilities, which can realize collaborative solutions to problems, and at the same time use the high-speed computing capabilities of calculators to find efficient and fast optimal solutions.
2. Nonlinear processing: as the brain thinking is nonlinear, the neural network working method that imitates human thinking must also be nonlinear, so that the calculator has an advantage in solving nonlinear-related problems.
3. Automatic learning function: by imitating the human brain management method, a special neural network model is developed, which can combine the data in the existing data.
4. Ambiguous calculation ability: for problems that cannot be accurately established in the real world, traditional calculation methods seem powerless, but the human brain can effectively solve problems.

At present, among the abnormal behavior testing algorithms based on neural networks, shallow neural network algorithms have been widely used. But the BP algorithm is particularly prominent. BP has learned this algorithm, and its algorithm is as follows:

1. Initialization center: the weight and bias of the neural network are first reconciled into a relatively small random number.
2. Forward input: when calculating the output of each unit in the sedimentary layer and the output layer, *w* is the weight of the right layer neuron, *o* is the output of the upper neuron, and *A* is the neuron.
3. Back propagation: by updating the weights and deviations, errors in the network learning process will propagate backwards. Through the periodic operation of forward rafter input and back propagation error, it becomes the deviation value of *A* and back propagation.

In order to successfully determine the classification of the support vector machine and the weighted KNN algorithm, this paper has carried out a detailed inspection of the algorithm and verified whether the result is more suitable than the traditional SVM-KNN algorithm. Data classification algorithms are usually used in classification, and text classification is widely used in data collection, AI speech, and the Internet. In this chapter, the developed weighted KNN algorithm is combined with the development of LIBSVM, which is relatively widely used for data classification in learning.

The accuracy of this article is mainly divided into two categories: test data text classification and UCI. The text from the three UCI database data collected from the data is classified into the internal load, and the data of the accurate target text of the result is classified. In this case, determine the range of *k* of the classifier that keeps the two types in the algorithm and the accuracy of the result distinguishing all text data types with the same value, classifying records by type. In the two experiments, the SVM and KNN algorithms used were separated from the traditional SVM-KNN algorithm and statistical set, and the accuracy was compared with the difference in classification results, as shown in Table 2.

Among them, TP is inferred from positive points as positive points, TN infers negative points as negative points, FP infers negative points as positive points, and FN infers positive points as negative points. The parameter formula is

(27) $$\begin{array}{c} \text{accuracy}=\frac{\text{TP}+\text{TN}}{\text{TP}+\text{TN}+\text{FP}+\text{FN}}*100\%. \end{array}$$

The recall rate is the ratio of the accurate sample of the sample to the relevant type of sample. The reference formula is as follows:

(28) $$\begin{array}{c} \text{recall}=\frac{\text{TP}}{\text{TP}+\text{FN}}*100\%. \end{array}$$

The accuracy is estimated based on an accurate sample, and all guesses are based on the proportion of the correct sample. The formula for the parameters is as follows:

(29) $$\begin{array}{c} \text{precision}=\frac{\text{TP}}{\text{TP}+\text{FP}}*100\%.s. \end{array}$$

The *F*1 value is the harmonic mean, and the parameter formula is

(30) $$\begin{array}{c} F1=2*\frac{\text{precision}*\text{recall}}{\text{precision}+\text{recall}}\\ =\frac{2\text{TP}}{2\text{TP}+\text{FP}+\text{FN}}*100\%. \end{array}$$

In order to ensure that the classification results of the calculation methods are objective and accurate, in this section, the classification results of the calculation methods are verified by two different data collections: Chinese language downloaded from the laboratory and machine learning in the UCI database. SPECTF heart, Transfusion, Ionosphere, and Diabetes from the UCI machine learning database use the text classification database to contain a large amount of data and information on the Internet. The entire material library includes several text classifications. First, choose a sample that balances the flow. About 2,000 text samples of each type are used as training manual data samples, and 200 samples of each type are selected as the samples to be measured. Secondly, an unbalanced sample set was selected. Among the 2000 samples of each type, a randomly numbered text sample is used as the training set, and the remaining samples are estimated with unknown samples, as shown in Table 3.

UCI data collection experimental data processing method: data classification is performed through the four types of data sets: SPECTF heart, Transfusion, Ionosphere, and Diabetes from the UCI machine learning database to test the classification accuracy of the improved SVM-KNN hybrid classifier, and the classification accuracy of the SVM-KNN hybrid classifier is improved.

Aiming at the classification performance problem between the weighted KNN algorithm and the traditional KNN algorithm, the difference in the classification accuracy of the two algorithms is verified through experiments, and the accuracy between the weighted KNN algorithm and the existing KNN is improved through Iris data set.

The data of this experiment are taken from the Iris data set in the UCI database. The total number of samples in the Iris data set used is 300, which contains 3 categories. The number of samples of each type is different, which may lead to unbalanced data distribution, and the classification results of the traditional KNN algorithm are compared and verified, as shown in Table 4.

Two-thirds of the data of A, B, and C in the Iris data set are randomly selected and used for training data collection, and the remaining data are used for test data collection. The following table is part of the Iris data set data, as shown in Table 5.

Therefore, in order to obtain a weighted KNN and existing KNN algorithm to accurately control the classification results of the imbalanced data set, through the data classification experiment on the Iris data, observe that when different *K* values are taken, the two algorithms are classifying the difference in accuracy. Based on the above laboratory example, the sample value predicted by Iris is used for category prediction, and Matmart Simulator classifies the two calculation methods into different *K* values based on different *K* values. The error rate is summarized as follows: in order to classify more intuitively, you must note the relationship between the error rates of the classification results, as shown in Table 6.

It can be seen that the weighted KNN algorithm developed when K is small has higher numerical classification accuracy than the traditional KNN algorithm. Because the accuracy is incorrect, the weighted KNN calculation method can be obtained by adding an adjustment factor. Compared with the existing KNN classification effect in the unbalanced set, the classification accuracy is improved to a certain extent, thereby improving the classification accuracy.

## 4. Forecast of Internet Digital Economy Development

### 4.1. The Connotation, Development Significance, and Advantages and Difficulties of the Digital Economy

From the perspective of digital economy technology and its development process, digital economy is the inevitable product of the development and dissemination of Internet technology and the in-depth implementation of the “Internet +” development strategy. The development of the digital economy has become a new starting point, driving the growth rate of the Chinese economy to exceed a moderately high level. Innovatively develop technology industries, imitate independent innovation and development, and develop a traction that is sufficient to show the digital economy. This is an important innovative strategic force to develop and change China's social and economic development. The digital economy is a mixed development industry with additional functions. It has unique functions in optimizing industrial resource allocation, coordinating industrial structure and transformation, and improving efficiency while ensuring the quality of traditional manufacturing products and services. At present, China's economy has entered a new stable state. Ineffective supply and low-cost supply are important obstacles to economic development and transformation. The digital economy helps the integration of agriculture, industry, service industries, and the Internet, through innovating business models and management methods, improving management efficiency and organizational efficiency, accelerating the development of traditional enterprise forms, and tailoring production according to market demand, expanding the effective supply of the market.

The rapid development of China's digital economy is due to its own internal advantages, in addition to finding new economic development impetus in a new stable state of economy. First of all, this is because China's Internet distribution is relatively good, and a large number of Internet users have laid the market foundation for China's digital economy. Currently, China has the largest broadband network in the world, including almost all cities. The transformation of traditional companies is inevitably limited by the two key factors of capital and technology. At the same time, the digital technology in the digital economy model has produced a series of social problems, which come from rural areas. During the application process, it will bring many challenges and drawbacks to the development, and there are problems with the rapid development of the digital economy and the relatively backward laws and regulations. At the same time, the Internet of Things in the digital economy model is also subject to greater security threats than the Internet. Because software and hardware work at the same time, many parts of the Internet of Things and its components cannot be resolved through upgrades, revisions, replacements, etc., and must be addressed. Complete restoration is therefore more difficult and potential losses are greater. In addition, due to the vast territory of China, there are also digital divide issues in the development of digital economy, including digital divides between regions and digital divides between classes.

### 4.2. Development of China's Internet Digital Economy

The digital economy development model is a concentrated expression of satisfying the five ideologies of “innovation, harmony, green, openness, and sharing,” and is an important driving force and means to change the current social economic operation and development model. In the process of development, the systems, models, and theories of traditional economic models will inevitably change to reveal a new social economic form. Therefore, it is necessary to ensure the healthy development of China's digital economy. At the same time, in the process of social and economic transformation, enterprises and governments will be the direct participants in the reform, so it is also necessary to pay attention to the digital transformation of enterprises and the digital transformation of governments.

### 4.3. Recommendations for Promoting the Healthy Development of China's Digital Economy

According to foreign experience in the development of digital economy and the actual obstacles to the development of China's digital economy, the development of the digital economy and society will face many problems, especially the labor distribution driven by artificial intelligence, which can be reflected in the AI economy. Internet technology is the development category of the digital economy. Due to the rapid development of the basic Internet technology of the digital economy and the innovative capabilities of the digital economy, it will inevitably lead to the lag and inapplicability of traditional regulatory laws. It is necessary to strengthen the legal system of the digital economy, to ensure the timeliness of relevant laws and regulations. At the same time, it is necessary to break through the protection barriers of the digital growth era and the regional protectionism of the existing economic model, fully realize the universal concept of the digital economy era, and accelerate the development of the digital economic model. In addition, the development of the digital economy model is a systematic project that requires the joint efforts of the entire society. Therefore, it is also necessary to improve the digital literacy level of the whole people, which can be achieved through vocational skills training, competitions, and training camps.

### 4.4. Enterprise Digital Transformation and Development

At present, digital technology is constantly innovating traditional economic and business models, and new business models have caused the boundaries of traditional industries to continue to blur, and the frequency of interaction between industries continues to increase. Therefore, accelerating the digital transformation of traditional enterprises is what makes enterprises competitive. It is not only an inevitable requirement for maintaining vitality, but also an inevitable requirement for the development environment outside of the socioeconomic environment. From the experience of international enterprise digital development and transformation, the best way for enterprise digital development is as follows: directly establish a primitive ecological digital company. Through the integration and development of traditional technology enterprises, we will accelerate the transnational cooperation of enterprises and promote the digital transformation and development of traditional enterprises. The development of digital transformation within traditional enterprises is an enterprise development plan from various angles such as corporate culture and marketing. Through the brand to ensure the company's competitive advantage in the market and by ensuring the consistency of online and offline services, a close connection between online and offline consumption is achieved. Online is mainly product selection and customization, and offline is mainly product experience and after-sales.

### 4.5. Government Digital Transformation

It is recommended that government departments, as the makers and executors of digital economy development policies, determine the direction and quality of the digital economy development of the entire society to a certain extent through their digital management capabilities, the “Internet +” action plan, the development of the sharing economy, and the national big data strategic thinking. In addition, the digital government also realizes the content innovation and model innovation of traditional government activities through service innovation and management innovation. Therefore, the government's digital transformation must be carried out from two aspects: model and system, and at the same time it have to strengthen the government's data sharing and openness, ensure that data resources are fully utilized, and ensure that the intrinsic value of data resources is fully released. Through the establishment of a data market application mechanism, the participation of all employees is mobilized, and a market transaction mechanism is introduced to develop and use data information. In addition, a data security and privacy protection system has also been established, especially that the government has established a digital exit mechanism to ensure data security.

## 5. Conclusion

In order to solve the neural network model based on word and phrase division, this paper introduces the structure of the neural network anomaly detection classification algorithm based on the character level in detail and designs the overall architecture diagram of the intrusion detection system using the character-level algorithm. Finally, in the actual network data collection, the intrusion detection model is designed and verified by experiments and compared and analyzed with other intrusion detection models. According to the experimental results, the neural network detection algorithm based on character level proposed in this paper can clearly show its detection rate, false report rate, recovery rate, and accuracy. In order to improve the classification performance of classification calculation methods, people need to research and develop various calculation methods. Based on the related literature research of SVM-KNN classification algorithm and KNN classification algorithm, according to the classification characteristics of the current KNN classification algorithm and the combination of the advantages of the harmonic weighted KNN algorithm and the support vector machine algorithm, the combination of SVM and KNN algorithm will be combined to construct a harmonic weighted KNN algorithm based on SVM to improve the classification performance of the data set.

By comparing the classification results with the traditional SVM-KNN classifier, it is concluded that the classification performance of the HWSKNN classifier on the imbalanced data set has been improved. The experimental results show that the character-level neural network detection algorithm proposed in this paper is obviously due to other detection algorithms in terms of detection rate, false alarm rate, recall rate, and accuracy. In summary, there are three purposes and significance of neural network applications in the field of intrusion detection:

1. The introduction of convolutional neural networks into intrusion detection systems can improve detection accuracy and efficiency and solve key problems in the field of network security
2. The application of neural network algorithms based on character sets to intrusion detection is in the field of deep learning, and also there is a new attempt in the field of intrusion detection, which can be used to deepen the understanding of the characteristics and relationships of network intrusion data
3. Promote the application of neural networks in other research fields besides image processing and speech recognition

The main results and research innovations of this paper are as follows: 1. Because the support vector algorithm has good classification performance when it is far away from the interface, most of the classification errors are concentrated in the area around the interface. Under the ideal data set distribution, applying the KNN classifier to the data distribution around the interface helps to improve the classification accuracy. 2. Based on the research of the weighted KNN algorithm in the past, a new improved method is proposed for the cumbersome parameter selection of the existing calculation and distribution and the partial minority prediction problem of unbalanced data, and the classification of the algorithm is appropriately adjusted by adding an adjustment factor to the minority class problem.

## Figures and Tables

**Table 1:** Comparison of traditional machine algorithms.

Algorithm	Algorithm description	Advantage
Logistic regression	Softmax function is used as a multiclassifier.	The amount of calculation is relatively small and easy to understand and implement.
Support vector machines	Find the optimal hyperplane to determine the classification category *f* (*x*) = sign(*w* · *x* + *b*).	It can handle the interaction of nonlinear features, solve high-dimensional problems, and improve generalization ability.
Naive Bayes	The highest posterior probability of the output category is used as the input target category: *P*(*y*_*i*_/*x*)=*p*(*x*/*y*_*i*_)*·p*(*y*_*i*_)/*p*(*x*)	Stable classification efficiency and good performance on small-scale data. It is less sensitive to lost data.
K neighbors	Select the KNN feature spaces of the nearest category, and input samples as output.	It is easy to use and understand and can be used for nonlinear classification.

**Table 2:** Classification evaluation index.

	Positive	Negative
True (correct guess)	True positive (TP)	True negative (TN)
False (wrong guess)	False positive (FP)	False negative (FN)

**Table 3:** Balance text categories and quantity.

Text type	Number of texts (pieces)
Car	2000
Physical education	2000
Tourism	2000
Education	2000
Culture	2000

**Table 4:** Data set description.

Data set category	Number of samples	Percentage of samples	Category number
Iris-setosa	50	16.6	A
Iris-versicolor	100	33.3	B
Iris-virginica	150	50	C

**Table 5:** Part of the Iris data set data.

Category	Calyx length (cm)	Calyx width (cm)	Petal length (cm)	Petal width (cm)
A	5.2	3.5	1.6	0.3
A	4.8	2.9	1.5	0.2
B	7.1	3.3	4.8	1.4
B	6.8	3.1	5.0	1.5
C	6.3	2.9	5.7	2.2
C	6.4	3.1	5.9	2.3

**Table 6:** Error rate of the two algorithms.

K	10	20	30	40	50
Traditional KNN	0.075	0.065	0.172	0.167	0.257
Improved weighted KNN	0.050	0.044	0.156	0.169	0.253

## Data Availability

The data used to support the findings of this study are available from the corresponding author upon request.
